# Cardiovascular Toxicity of Tyrosine Kinase Inhibitors Used in Chronic Myeloid Leukemia: An Analysis of the FDA Adverse Event Reporting System Database (FAERS)

**DOI:** 10.3390/cancers12040826

**Published:** 2020-03-30

**Authors:** Santa Cirmi, Asmae El Abd, Louis Letinier, Michele Navarra, Francesco Salvo

**Affiliations:** 1 Department of Chemical, Biological, Pharmaceutical and Environmental Sciences, University of Messina, I-98168 Messina, Italy; scirmi@unime.it; 2 Inserm, UMR 1219, Team Pharmacoepidemiology, Bordeaux Population Health Research Center, University of Bordeaux, F-33000 Bordeaux, France; asmaeelabd2@gmail.com (A.E.A.); louis.letinier@u-bordeaux.fr (L.L.); francesco.salvo@u-bordeaux.fr (F.S.); 3 Service de Pharmacologie Médicale, Pôle de Santé Publique, CHU de Bordeaux, F-33000 Bordeaux, France

**Keywords:** tyrosine kinase inhibitors, cardiovascular toxicity, chronic myeloid leukemia, FAERS, adverse drug reaction

## Abstract

Tyrosine kinase inhibitors (TKIs), the treatment of choice for chronic myeloid leukemia (CML), can be associated to cardiovascular (CV) adverse events (AEs). A case/non-case study was performed using AE reports registered in the Food and Drug Administration (FDA) Adverse Event Reporting System (FAERS) database to compare the risk of CV event reports related to TKIs indicated in the management of chronic myeloid leukemia (CML). Disproportionality of CV event-related TKIs was computed using the Reporting Odds Ratio (ROR) as a measure of potential risk increase. Nilotinib accounts for more than half of reported cases related to TKIs. Signal of Disproportionate Reporting (SDR) was found for cardiac failure, ischemic heart disease, cardiac arrhythmias, *torsade de pointes*/QT prolongation, hypertension, and pulmonary hypertension. Dasatinib and bosutinib were related to the highest disproportionality for cardiac failure. Nilotinib was associated with the highest SDR for ischemic heart disease, *torsade de pointes*/QT prolongation and cardiac arrhythmias. Only ponatinib was related to an SDR for hypertension, while dasatinib and imatinib were related to pulmonary hypertension. In the context of CML, TKIs have different safety profiles related to CV events, among which nilotinib seems particularly related to. These results claim for a revision of its CV safety profile mainly for the risk of *torsade de pointes*/QT prolongation.

## 1. Introduction

Chronic myeloid leukemia (CML) is a malignant disease of haemopoietic stem cells with high incidence (1–2 cases per 100,000 adults) without any major geographic or ethnic difference [1]. The average age at diagnosis ranges from 60 to 65 years in Europe. The prevalence of CML is steadily rising due to the substantial prolongation of survival that has been achieved with targeted therapy [2]. CML arises from a chromosomal translocation causing a shortened chromosome 22 designated as the Philadelphia chromosome. This translocation leads to a fusion of the ABL1 and BCR genes that codes for the BCR-ABL1 protein characterized by high tyrosine kinase activity [3]. Therefore, efforts were aimed at developing compounds that could selectively inhibit the aberrant tyrosine kinase resulting from this molecular rearrangement. 

Imatinib was the first tyrosine kinase inhibitor (TKI) approved by the FDA for the treatment of patients with CML. It dramatically increased the 5-year overall survival rate from 11% to 90% among Philadelphia chromosome-positive patients with CML [4]. Since then, TKIs were considered as the treatment of choice in CML, and several others have been developed. Although all approved TKIs have activity against BCR-ABL1, they are different in their potency and activity against BCR-ABL1. Second-generation TKIs, initially developed to overcome imatinib resistance, were subsequently shown to induce more rapid and profound molecular responses; actually, along with imatinib, nilotinib, dasatinib and bosutinib were approved for front-line therapy [5,6,7]. Ponatinib, a third-generation TKI, is the only clinical TKI active against the BCR-ABL1^T315I^ mutation [8,9]. It was initially approved in the United States with a fairly broad label, but after reports of cardiovascular toxicity, its indication was restricted to patients with the BCR-ABL1^T315I^ mutation or in whom other TKIs were not indicated [10].

These events raised the attention on cardiotoxicity and cardiovascular (CV) adverse events related to TKIs, as cardiomyopathy, cardiac failure, acute coronary syndromes or peripheral artery disease [11], which could be even life-threatening or fatal. Thus, they can lead to life-saving interventions, discontinuation of TKI treatment or dose reduction, leading to poor treatment responses and reduction of survival rate in these patients.

Spontaneous adverse reaction reports, such as those available in the United States Food and Drug Administration (FDA) Adverse Event Reporting System (FAERS) database, permit analysis of a big quantity of data for safety signals. FAERS contains real-world results from a large population and under conditions that may have been overlooked in controlled studies, which lack the ability to detect the whole spectrum of adverse drug reactions. Therefore, considering these aspects, FAERS, as well as other spontaneous reporting systems, represents a precious resource for clinicians, regulators and pharmaceutical companies to promptly detect new associations, assess the risk-benefit profile of drugs over time and finally support safe prescribing of the drugs.

The aim of this study was to compare the risk of reporting CV events in patients treated with TKIs indicated in the management of CML, and to describe the clinical characteristics of the involved patients. 

## 2. Results

### 2.1. Descriptive Analysis

Among the 20,204,802 reports recorded in FAERS in the study period, a total of 1,306,242 AEs reports were related to anticancer drugs. After exclusion of nonserious AEs (163,354 reports), duplicate cases (357,517 reports), and aberrant cases (68,208 reports), 717,163 reports were included in the present analysis (Figure S1).

A total of 64,232 cases of CV events were reported and, among these cases, 3930 (6.1%) were related to TKIs (Table 1). The median age of TKIs cases was 65 years (Q1:55, Q3:73), while patients exposed to other anticancer drugs were slightly younger (63 years, Q1:52, Q3:71). In TKI cases, the rate of men was higher than women (52.9% vs. 36.2%; in 10.9% the sex of the involved patient was not reported), while in non-cases this difference was less important (48.5% vs. 41.7%; 13.5% not reported). Cases exposed to TKIs were in majority reported by healthcare professionals (73.2%), and mainly from the United States (32.1%), Japan (10.6%), and France (7.5%). In most reports, the TKI was considered as the suspected drug in the CV event occurrence (83.2%). Hospitalizations accounted for 35.0% of cases, while 10.1% of the cases had a fatal issue; in non-cases, the rate of fatal events was 17.4%.

Concerning active principles, for a total of 3930 CV events reported for TKIs, 59.0% were related to nilotinib, 21.2% to dasatinib, 14.4% to ponatinib, 4.4% to imatinib and 1.0% to bosutinib (Table 2). CML was the indication of use in 68.1% of cases, while Acute Lymphocytic Leukemia (ALL) and Gastrointestinal Stromal Tumours (GISTs) were the indication of use in 4% and 1.3% of cases reports, respectively. We noted a global trend in the increase of the number of cases with time (3.1% in 2008 vs. 17.3% in 2018), but a similar trend was also noted for non-cases.

### 2.2. Disproportionality and Time-to-Onset Analyses

Six of the eight selected Standardized MedDRA Queries (SMQs) were reported with more frequency with TKIs than with other anticancer drugs, notably—cardiac failure (adjusted Reporting Odds Ratio (aROR) 2.4; 95% CI: 2.2–2.6), ischemic heart disease (3.8; 3.6–4.1), cardiac arrhythmias (1.7; 1.4–2.1), *torsade de pointes*/QT prolongation (6.6; 5.6–7.8), hypertension (1.2; 1.0–1.4), and pulmonary hypertension (3.9; 3.2–4.7; see Tables S1–S8 for details of crude and aROR and case numbers). 

**Figure 1:**
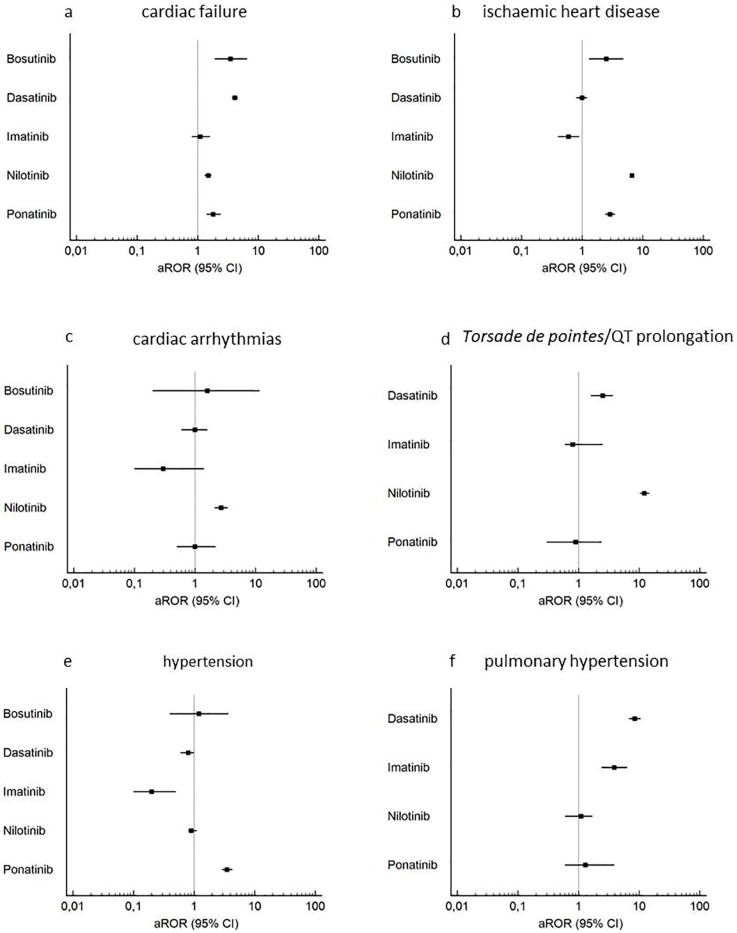
Forest plots of disproportionality (aROR) of TKIs and cardiovascular events.

**Figure 2:**
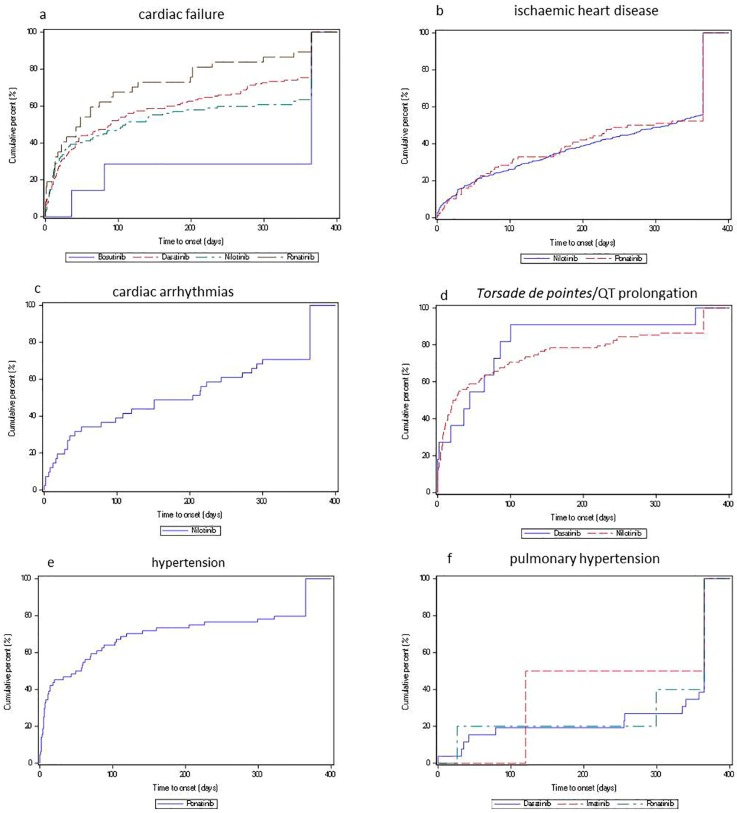
Cumulative distribution function of TKIs by time-to-onset.

Concerning cardiac failure, a significant increased aROR was found for all analysed TKIs, with the exception of imatinib (aROR 1.1, 0.8–1.6; 51 cases). Dasatinib was related to the highest ROR (4.1, 3.7–4.6; 363 cases), followed by bosutinib (3.5, 1.9–6.6; 12 cases,), while for all the other TKIs the aROR was lower than two (Figure 1a). The median time-to-onset for dasatinib was 89.5 days (Q1:17, Q3:349), for nilotinib was 110 days (Q1:11, Q3:365), and for ponatinib was 48 days (Q1:13, Q3:199) (Figure 2).

Concerning ischemic heart disease, a significantly increased aROR was found nilotinib (6.7, 6.2–7.2; 1243 cases), ponatinib (2.9, 2.4–3.5; 154 cases), and bosutinib (2.5, 1.3–4.8; 11 cases, Figure 1b). The median time-to-onset for nilotinib was 318 days (Q1:91, Q3:365), and for ponatinib was 280.5 days (88 cases, Q1:75, Q3:365) (Figure 2).

Concerning cardiac arrhythmias, only nilotinib was associated with a significantly increased aROR (2.7, CI 95% 2.1–3.5; 99 cases, Figure 1c) for a median time-to-onset of 204 days (Q1:32, Q3:365). Regarding *torsade de pointes*/QT prolongation, nilotinib (12.2, 10.3–14.6; 248 cases), and dasatinib (2.5, 1.6–3.7; 36 cases,) were associated with an increased aROR (Figure 1d). The median time-to-onset for nilotinib was 23 days (Q1:6, Q3:138), and for dasatinib was 44 days (Q1:2, Q3:83) (Figure 2). 

Concerning hypertension, only ponatinib was related to a significant aROR (3.5, CI 95% 2.9–4.3; 134 cases, Figure 1e) with a median time-to-onset estimated at 53 days (Q1:5.5, Q3:215.5). Concerning pulmonary hypertension, significant aRORs were found for dasatinib (8.5; 6.8–10.6; 113 cases), and imatinib (3.9; 2.4–6.4; 19 cases, Figure 1f), with a median time-to-onset of 365 days (26 cases; Q1:256, Q3:365) for dasatinib, while only two cases were informative for imatinib (Figure 2). 

## 3. Discussion

It is known that some anticancer drugs are cardiotoxic and the importance of this is being increasingly recognized. In addition to sharing risk factors, the aging of the general population in developed countries makes it more probable that a patient has both cancer and cardiovascular disease, which may present a considerable therapeutic challenge and has a significant impact on the overall prognosis and survival of cancer patients. 

TKIs represent the standard therapy for patients with CML. However, there are differences in the safety profiles of each TKI with reference to cardiovascular toxicities, which occur more often with chronic treatment. Moreover, because the median age of diagnosis for CML is 64 years, these older patients may have comorbidities and risk factors that increase the TKI-mediated toxicities during long-term treatment. In line with the literature, the present study indicates that TKIs used in CML have a different safety profile regarding CV events, maybe due to different target profiles of these TKIs that contribute to their distinct toxicity profiles [12,13]. 

The present study has some limitations. The most classic weakness of spontaneous reporting is under-reporting; in routine pharmacovigilance, the reporting rate is on average 6% of the actual cases of adverse effects. Moreover, under-reporting could be selective and more massive for nonserious cases [14,15]. It is thus likely that the present results strongly underestimated the number of cases of CV events occurring in patients exposed to TKIs; even the number of reported cases was relatively high in FAERS. Another limitation of this study is due to a lack of data concerning the line of therapy. From a general point of view, serious adverse events could occur more frequently in patients with advanced phases of their disease. For the present study, this could imply a potential overestimation of the association between CV events and TKIs used as second-line (e.g., dasatinib), rather than those ones used as first-line. Nevertheless, this limitation does not probably concern molecules given as second-line treatments, which should be done a priori to patients with similar background risk. 

The first finding of this study concerned imatinib, as it seems less related to CV events than other TKIs used in the management of CML patients. Imatinib was related to pulmonary hypertension with a relatively low number of cases. This could be related to the study period (2008–2018), which do not include the first years of imatinib use, as it was marketed in 2002. Nevertheless, as cardiac events related to TKIs became an issue with time passing [16], this apparent limitation does not seem to be a major issue in this study. Except imatinib, all the TKIs were related to cardiac failure, but the magnitude of this association was different among drugs, and this result is in accordance with the literature [17]. In particular, bosutimib and dasatinib showed the highest values of aROR, which merit further investigation in order to confirm or rule out a putative comparative increased risk. 

Concerning the different CV events analysed, the only acute event was *torsade de pointes*/QT prolongation, while the other ones seemed to be more retarded events, as the dose was more frequent after three months (i.e., cardiac failure), or around one year (i.e., ischemic heart disease and pulmonary hypertension).

In this study, nilotinib was particularly related to *torsade de pointes*/QT prolongation with the highest values of aROR, and a relatively high number of cases. Moreover, it was the only TKI associated with a significant increase in the reports related to cardiac arrhythmia. Nilotinib is already known as a drug with a possible risk of *torsade de pointes* [18]. The present data suggest a revision of the evidence of *torsade de pointes* related to nilotinib that could imply consideration of nilotinib as a drug at definite risk of *torsade de pointes*/QT prolongation. Nilotinib showed also high disproportionality for ischemic heart disease, maybe due to the inhibition of the tyrosine kinase activity of the PDGF and c-Kit receptors in addition to that of BCR-ABL [19]. 

Our data are in line with those presented in literature showing an increased incidence of CV events in patients treated with nilotinib compared to other TKIs. For instance, the results of the ENESTnd prospective randomized study showed that the incidence of cardiovascular events after 6 years was 10% in patients treated with nilotinib (5% ischemic heart disease, 1.4% ischemic cerebrovascular disease, and 4.3% peripheral arterial disease) as compared to 2.5% in imatinib-treated patients [20]. Consequently, current experts’ recommendations advocate against the use of nilotinib in patients with a high-risk cardiovascular profile, whenever possible [21].

Dasatinib was initially approved for salvage treatment and subsequently for front-line CML therapy, based on superior 12 month complete cytogenetic response rates compared with imatinib [22]. The nonhematologic safety profile was similar to imatinib with the exception of frequent pleural effusions [23]. In October 2011, the FDA issued a warning regarding cardiopulmonary risks of dasatinib and recommended that patients be evaluated for signs and symptoms of cardiopulmonary disease before and during dasatinib treatment [24]. In our study, we found that dasatinib was related to *torsade de pointes*/QT prolongation (with a relatively low number of cases) and pulmonary hypertension. Both these findings are well-known and do not allow particular actions. Conversely, we did not find any disproportionality for ischemic heart disease, despite also dasatinib inhibits PDGF and c-Kit receptors [25]. This could be due to a form of masking bias between this event and other events [26,27], as pulmonary arterial hypertension, which could be relatively more frequently reported, as related to specific drug agency alerts [24].

Ponatinib was designed to inhibit BCR-ABL1^T315I^ and was approved after encouraging results from a phase II study [9]. In vitro profiling revealed potent inhibition of numerous tyrosine kinases including VEGFR1-3 [11] which could explain the high disproportionality for hypertension observed. In the PACE study, 26% of patients developed hypertension, which was predictable given the VEGFR2 inhibition by ponatinib [28]. To date, some “real-life” studies concerning ponatinib have been published. In the PEARL study, the incidence of CV toxicity was slightly higher than in the phase 2 PACE study, particularly for hypertension that occurred in 19.3% of patients in the real-life setting [29]. Therefore, these results highlighted the necessity to improve the control of CV risk factors and patient selection prior the prescription of ponatinib in real-life clinical practice [30] 

Moreover, ponatinib was related to cardiac failure, embolic and thrombotic events (Table S4), and ischemic heart disease. These events share, at least in part, the same pathophysiology and, as for nilotinib, could be due to the capacity of its activity on PDGF and c-Kit receptors [31]. The present study suggests particular attention of clinicians in particular with patients treated on a long-term basis. 

In our study, bosutinib was related to SDRs of ischemic heart disease and cardiac failure. In both these events, the low number of cases could not allow any meaningful conclusion. Moreover, bosutinib is mostly used as second-line treatment in patients with resistant CML or who are intolerant to prior therapies [19], which could include more severe patients, with a higher risk of developing cardiac complications.

## 4. Materials and Methods

### 4.1. Data Source

The study was conducted using data from adverse event reports recorded in the publicly available version the United States Food and Drug Administration (FDA) Adverse Event Reporting System (FAERS) database.

FAERS is an important source of post-marketing safety surveillance for all approved drug and therapeutic biologic products in the US, and it consists of more than 20 million reports up to May 2018. Moreover, even though FAERS is an US database, it has worldwide coverage, receiving serious reports from EU and other non-US countries. Therefore, the size and worldwide coverage of this database makes it particularly robust for the conduction of spontaneous reporting data analysis. 

The database contains anonymized reports submitted by healthcare professionals, consumers, manufacturers and other sources (FDA Adverse Event Reporting System (FAERS)-FDA Adverse Event Reporting System (FAERS): Latest Quarterly Data Files). The data set of FAERS consists of seven data tables, in which demographic information about the patient (e.g., age, sex, weight), source and type of the report, country of the reports, drugs with dates of start and end (when available), doses and routes of administration, AEs and their outcomes, and indications of use were contained. 

In FAERS, AEs are coded using event-related information according to the Medical Dictionary for Regulatory Activities (MedDRA). MedDRA is a hierarchical dictionary that could be used to code diagnoses, symptoms and signs, investigations, surgical and medical procedures, as well as therapeutic indications, and medical/social history [32,33].

In order to select the CV events of potential interest for this study, the SMQs available were screened (MedDRA Maintenance and Support Services Organization. Introductory Guide for Standardised MedDRA Queries (SMQs) Version 15.0. Chantilly (VA): International Federation of Pharmaceutical Manufacturers and associations; 2012). SMQs group all the terms representing signs, symptoms, investigations or diagnoses likely to be relevant to a defined medical condition or area of interest.

Most SMQs have two different types of search—the narrow search is composed of terms that are without any reasonable doubt related to a selected event; the broad search includes terms of the narrow one and terms that could be related to an event of interest, but for which there exists a degree of uncertainty. Thus, the narrow search is intended to be more specific, while the broad one is intended to be more sensitive. A previous work in the French Pharmacovigilance database showed that the narrow version of four different SMQs is equivalent to the broad version in terms of sensitivity, but have globally better performances in terms of positive predictive value of case identification [34]. Thus, for this study narrow version of the selected SMQs was used.

### 4.2. Study Design

A cross-sectional case/non-case study was performed in a subset of the FAERS database containing reports of serious events collected between April 1, 2008, and December 31, 2008, which included at least one anticancer drug among suspect, interacting or concomitant drugs. Cases were identified as reports with at least one of the following CV events as retrieved using the following SMQs—cardiac failure, cardiomyopathy, hypertension, pulmonary hypertension, ischaemic heart disease, *torsade de pointes*/QT prolongation, cardiac arrhythmias, embolic and thrombotic events (see Tables S9–S16 in supplementary materials). On the contrary, all other reports with serious AEs were considered as non-cases. For example, when reports with cardiac failure were considered as cases, all other serious reports were considered as non-cases, including reports with other CV events.

For this study, only TKIs which had a European marketing authorization for CML were considered, namely—imatinib, dasatinib, bosutinib, nilotinib, ponatinib. The list of these drugs was derived from European Society for Medical Oncology (ESMO) Clinical Practice Guidelines [3].

### 4.3. Extracted Variables

For this study, the following data were retrieved from FAERS—the patient’s sex and age at the time of adverse event, the type of reporter (health professional or not), reporting year and country, the event, as well as its occurrence date, seriousness criteria and the outcome of the event. The following variables concerning TKIs were also extracted—drug name, drug role in event occurrence (suspect drug, concomitant or interacting), and prescription dates when available.

In the database used for this analysis, a line corresponded to a drug/ADR combination (thus several lines for a report that includes several drugs, and concern several ADRs). In FAERS, the drugs are reported as free text, either brand name or generic name can be reported, but also a combination of both, and misspellings can be present because of a lack of drug codification [20]. Therefore, a drug name archive including all generic and trade names of drugs marketed in the US was created using a public list available on the FDA websites—Drugs^@^FDA Data Files. The FAERS database was also a source for a reference list based on adverse event reports. This list contains all generic names of drugs that were notified to the FDA between June 1, 2014 and December 30, 2018 and their International Nonproprietary Name (INN). This mapping approach allowed allocation of a substance name to about 90% of all records in the entire database, the rest has been completed manually. Then, INN was indexed according to the ATC classification to select anticancer drugs (ATC code: L01, Antineoplastic Agents).

A major problem in spontaneous reporting data is the presence of duplicates (i.e., the same report submitted by different sources) and multiple reports (i.e., a follow-up of the same case with additional and updated information). In the present study a two-step procedure of deduplication was applied. Firstly, only the last version of cases for which a follow-up was available was used. Secondly, cases with the same event, event date, age, gender, and country of origin were considered as duplicated [35]. The reports in which the reported date of the start of a drug was after the date of the AE were considered aberrant and excluded from the analysis. 

### 4.4. Data Analysis

#### 4.4.1. Descriptive Analysis

A descriptive analysis of cases and non-cases exposed to TKIs was performed. The baseline characteristics were described by the usual parameters—mean and standard deviation for continuous variables and numbers and percentages for categorical variables. The calculation of the time-to-onset of CV events was carried out according to the following formula—(Time-to-onset = Event onset date–Therapy start date). The median and interquartile ranges were used to describe the time to onset.

#### 4.4.2. Statistical Analysis

The ROR was used as a measure of disproportionality between cases and non-cases. In this study, ROR is the ratio of the odds of the number of CV events related to TKIs compared to those related to other anticancer drugs. Its confidence interval was calculated with an alpha risk of 5%. The analysis was run only considering coupled CV events/TKIs with at least 3 cases. RORs were calculated as crude or adjusted for age and sex on using logistic regression models. Adjusted RORs of CV events (and corresponding 95% confidence interval, 95% CI) for TKIs as a class and the different TKIs (imatinib, dasatinib, bosutinib, nilotinib, ponatinib) were computed. The cumulative distribution of the time-to-onset of CV events related to a significant aROR was presented graphically.

## 5. Conclusions

Overall, the present study indicates that TKIs used in CML have a different safety profile regarding CV events. Nilotinib seems particularly related to CV events, and these results claim for a revision of its CV safety profile, in particular on those concerning the risk of *torsade de pointes*/QT prolongation.

## Figures and Tables

**Table 1:** Characteristics of cases of cardiovascular toxicity.

Characteristics	TKIs *n* = 3930	Other Anticancer Drugs *n* = 60,302	Total *n* = 64,232
Patient’s age, years, median (Q1-Q3)	65 (55–73)	63 (52–71)	63 (52–72)
Patient’s age			
≤25 26–50 51–75 ≥ 76 Unknown	41 (1.0) 436 (11.1) 1729 (44.0) 490 (12.5) 1234 (31.4)	2878 (4.8) 7009 (11.6) 27,051 (44.9) 6615 (11.0) 16,749 (27.8)	2919 (4.5) 7445 (11.6) 28,780 (44.8) 7105 (11.1) 17,983 (28)
Patient’s sex			
Female Male Unknown	1423 (36.2) 2079 (52.9) 428 (10.9)	27,205 (45.1) 24,951 (41.4) 8146 (13,5)	28,628 (44.6) 27,030 (42.1) 8574 (13,35)
Type of reporter			
Health professional Nonhealth professional Unknown	2877 (73.2) 951 (24.2) 102 (2.6)	44,575 (73.9) 12,908 (21.4) 2819 (4.7)	47,452 (73.9) 13,859 (21.6) 2921 (4.5)
Reporting country			
United Sates Japan Germany France Other countries Unknown	1263 (32.1) 418 (10.6) 293 (7.5) 275 (7) 1505 (38.3) 176 (4.5)	21,841 (36.2) 5788 (9.6) 4415 (7.3) 4519 (7.5) 22,625 (37.5) 1114 (1.84)	23,104 (36.0) 6206 (9.7) 4708 (7.3) 4794 (7.5) 24,130 (37.6) 1290 (2)
Drug role in event occurrence			
Primary suspect drug Secondary suspect drug Concomitant Interacting	3269 (83.2) 611 (15.5) 40 (1.0) 10 (0.2)	29,869 (49.5) 19,588 (32.5) 10,745 (17.2) 100 (0.2)	33,138 (51.6) 20,199 (31.4) 10,785 (16.8) 110 (0.2)
Outcome of event			
Death Life-threatening Hospitalization-initial or prolonged Disability Required intervention Congenital anomaly Other serious events	396 (10.1) 171 (4.3) 1375 (35.0) 63 (1.6) 2 (0) 1 (0) 1922 (48.9)	7920 (13.1) 3105 (5.1) 22,434 (37.2) 1056 (1.7) 150 (0.2) 14 (0) 25,623 (42.4)	8316 (12.9) 3276 (5.1) 23,809 (37.1) 1119 (1.7) 152 (0.2) 15 (0) 27,545 (42.9)
Cardiovascular toxicity, SMQs			
*Cardiac arrhythmias* *Cardiac failure* *Cardiomyopathy* *Embolic and thrombotic events* *Hypertension* *Ischemic heart disease* *Pulmonary hypertension* *Torsade de Pointes/QT prolongation*	126 (3.2) 669 (17.0) 78 (1.2) 1049 (26.7) 306 (7.8) 1306 (33.2) 127 (3.2) 269 (6.8)	1982 (3.2) 8402 (13.9) 3238 (5.4) 27,911 (46.3) 8236 (13.7) 8833 (14.6) 854 (1.4) 846 (1.4)	2108 (3.3) 9071 (14.1) 3316 (5.2) 28,960 (45.1) 8542 (13.3) 10,139 (15.8) 981 (1.5) 1115 (1.7)

Data are no. (%) of cases unless otherwise specified.

**Table 2:** Characteristics of cases of cardiovascular toxicity and non-cases submitted for tyrosine kinase inhibitors (TKIs).

Characteristics	Cases *n* = 3930	Non-Cases *n* = 20,443	Total * n = 24,373
Type of TKIs			
Bosutinib Dasatinib Imatinib Nilotinib Ponatinib	38 (1.0) 835 (21.2) 173 (4.4) 2319 (59) 565 (14.4)	220 (1.1) 6382 (31.2) 2291 (11.2) 9014 (44.1) 2536 (12.4)	258 (1.0) 7217 (29.6) 2464 (10.1) 11,333 (46.5) 3101 (12.7)
Reported indication			
Chronic myeloid leukemia Acute myeloid leukemia Acute lymphocytic leukemia Myeloid leukemia Gastrointestinal stromal tumour Hypertension Other indication Unknown	2678 (68.1) 16 (0.4) 159 (4.0) 22 (0.6) 52 (1.3) 19 (0.5) 341 (8.7) 643 (16.4)	12,450 (60.9) 150 (0.7) 1448 (7.1) 136 (0.7) 471 (2.3) 23 (0.1) 2376 (11.6) 3389 (16.6)	15,128 (62.1) 166 (0.7) 1607 (6.6) 158 (0.6) 523 (2.1) 42 (0.2) 2717 (11.1) 4032 (16.5)
Reporting year			
2008 2009 2010 2011 2012 2013 2014 2015 2016 2017 2018 Unknown	120 (3.1) 106 (2.7) 163 (4.1) 209 (5.3) 284 (7.2) 422 (10.8) 546 (13.9) 415 (10.6) 481 (12.2) 501 (12.8) 679 (17.3) 4 (0.1)	488 (2.4) 720 (3.5) 922 (4.5) 1157 (5.7) 1470 (7.2) 2042 (10.0) 2421 (11.9) 2023 (9.9) 2853 (14) 2803 (13.7) 3530 (17.3) 14 (0)	608 (2.5) 826 (3.4) 1085 (4.4) 1366 (5.6) 1754 (7.2) 2464 (10.1) 2967 (12.2) 2438 (10.0) 3334 (13.7) 3304 (13.6) 4209 (17.3) 18 (0)

Data are no. (%) of cases and non-cases. * Number of cases and non-cases.

## Supplementary Materials

The following are available online at https://www.mdpi.com/2072-6694/12/4/826/s1, Figure S1: Flow chart of the study population, Table S1: Disproportionality analysis of cardiac arrhythmias submitted for TKIs compared to other anticancer drugs, Table S2: Disproportionality analysis of cardiac failure submitted for TKIs compared to other anticancer drugs, Table S3: Disproportionality analysis of cardiomyopathy submitted for TKIs compared to other anticancer drugs, Table S4: Disproportionality analysis of embolic and thrombotic events submitted for TKIs compared to other anticancer drugs, Table S5: Disproportionality analysis of hypertension submitted for TKIs compared to other anticancer drugs, Table S6: Disproportionality analysis of ischemic heart disease submitted for TKIs compared to other anticancer drugs, Table S7: Disproportionality analysis of pulmonary hypertension submitted for TKIs compared to other anticancer drugs, Table S8: Disproportionality analysis of *torsade de pointes*/QT prolongation submitted for TKIs compared to other anticancer drugs, Table S9: Table with SMQ “Cardiac Failure” and PTs used, Table S10: Table with SMQ “Cardiomyopathy” and PTs used, Table S11: Table with SMQ “Hypertension” and PTs used, Table S12: Table with SMQ “Pulmonary hypertension” and PTs used, Table S13: Table with SMQ “Ischaemic heart disease” and PTs used, Table S14: Table with SMQ “*Torsade de pointes*/QT prolongation” and PTs used, Table S15: Table with SMQ “Cardiac arrhythmia” and PTs used, Table S16: Table with SMQ “Embolic and thrombotic events” and PTs used.
