# Supplementary material for: Cardiovascular Toxicity of Tyrosine Kinase Inhibitors Used in Chronic Myeloid Leukemia: An Analysis of the FDA Adverse Event Reporting System Database (FAERS)
Source: Cancers (Basel). 2020 Mar 30;12(4):826. doi: 10.3390/cancers12040826 (PMC7226142; doi:10.3390/cancers12040826)
Supplement: Supplementary file 1 [file cancers-12-00826-s001.pdf]

Supplementary Materials

Cardiovascular Toxicity of Tyrosine Kinase Inhibitors Used in Chronic Myeloid Leukemia: An Analysis of FDA Adverse Event Reporting System Database (FAERS)

Santa Cirmi, Asmae El Abd, Louis Letinier, Michele Navarra and Francesco Salvo

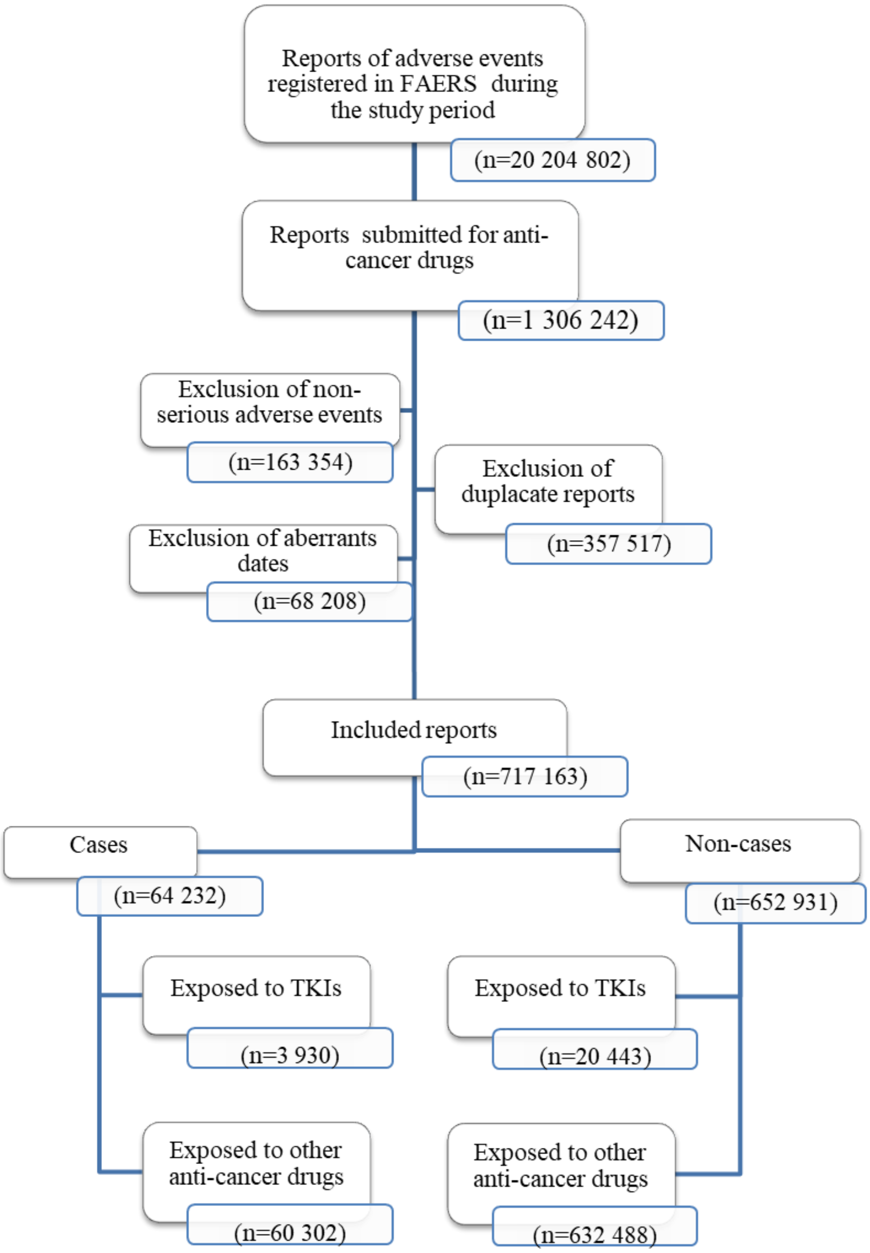

Figure S1. Flow chart of the study population.

Table S1. Disproportionality analysis of cardiac arrhythmias submitted for TKIs compared to other anti-cancer drugs.

Cardiac Arrhythmias				
**Drugs**	**Cases**	**Non-Cases**	**Crude ROR (95% CI)**	**aROR * (95% CI)**
All TKIs	142	52,483	1.7 (1.4–2.0)	1.7 (1.4–2.1)
Bosutinib	3	507	3.6 (1.1–11.6)	1.6 (0.2–11.7)
Dasatinib	23	14,351	1 (0.6–1.5)	1 (0.6–1.6)
Imatinib	5	5728	0.5 (0.2–1.3)	0.3 (0.1–1.4)
Nilotinib	99	24,345	2.5 (2.1–3.1)	2.7 (2.1–3.5)
Ponatinib	12	7552	1.0 (0.5–1.7)	1.0 (0.5–2.2)
Other anti-cancer drugs	2437	1,512,555	1 [Reference]	1 [Reference]

* adjusted for age and sex.

Table S2. Disproportionality analysis of cardiac failure submitted for TKIs compared to other anti-cancer drugs.

Cardiac Failure				
**Drugs**	**Cases**	**Non-cases**	**Crude ROR (95% CI)**	**aROR * (95% CI)**
All TKIs	744	51,881	2 (1.9–2.2)	2.4 (2.2–2.6)
Bosutinib	12	498	3.3 (1.9–5.9)	3.5 (1.9–6.6)
Dasatinib	363	14,011	3.6 (3.3–4.0)	4.1 (3.7–4.6)
Imatinib	51	5682	1.2 (0.9–1.6)	1.1 (0.8–1.6)
Nilotinib	242	24,202	1.4 (1.2–1.6)	1.5 (1.3–1.7)
Ponatinib	76	7488	1.4 (1.1–1.8)	1.8 (1.4–2.4)
Other anti-cancer drugs	10,545	1,504,447	1 [Reference]	1 [Reference]

* adjusted for age and sex.

Table S3. Disproportionality analysis of cardiomyopathy submitted for TKIs compared to other anti-cancer drugs.

Cardiomyopathy				
**Drugs**	**Cases**	**Non-cases**	**Crude ROR (95% CI)**	**aROR * (95% CI)**
All TKIs	105	52,520	0.5 (0.4–0.7)	0.5 (0.4–0.7)
Bosutinib	0	510	n/a	n/a
Dasatinib	25	14,349	0.5 (0.3–0.7)	0.4 (0.3–0.7)
Imatinib	21	5712	1.0 (0.7–1.6)	1.2 (0.7–2.0)
Nilotinib	41	24,403	0.5 (0.3–0.6)	0.4 (0.2–0.6)
Ponatinib	18	7546	0.7 (0.4–1.1)	0.6 (0.3–1.2)
Other anti-cancer drugs	5455	1,509,537	1 [Reference]	1 [Reference]

* adjusted for age and sex.

Table S4. Disproportionality analysis of embolic and thrombotic events submitted for TKIs compared to other anti-cancer drugs.

Embolic and thrombotic events				
**Drugs**	**Cases**	**Non-cases**	**Crude ROR (95% CI)**	**aROR * (95% CI)**
All TKIs	1294	51,331	0.9 (0.9–1-0)	0.9 (0.9–1-0)
Bosutinib	11	499	0.8 (0.4–1.5)	1 (0.5–1.9)
Dasatinib	194	14,180	0.5 (0.4–0.6)	0.5 (0.4–0.6)
Imatinib	66	5,667	0.4 (0.3–0.5)	0.4 (0.3–0.5)
Nilotinib	755	23,689	1.2 (1.1–1.3)	1.3 (1.1–1.4)
Ponatinib	268	7296	1.4 (1.2–1.6)	1.4 (1.2–1.6)
Other anti-cancer drugs	39,330	1,475,662	1 [Reference]	1 [Reference]

* adjusted for age and sex.

Table S5. Disproportionality analysis of hypertension submitted for TKIs compared to other anti-cancer drugs.

Hypertension				
**Drugs**	**Cases**	**Non-cases**	**Crude ROR (95% CI)**	**aROR * (95% CI)**
All TKIs	372	52,253	1.1 (1.0–1.2)	1.2 (1.0–1.4)
Bosutinib	4	506	1.2 (0.5–3.3)	1.2 (0.4–3.7)
Dasatinib	75	14,299	0.8 (0.6–1.0)	0.8 (0.6–1.0)
Imatinib	12	5721	0.3 (0.2–0.6)	0.2 (0.1–0.5)
Nilotinib	147	23,689	1.0 (0.8–1.2)	0.9 (0.8–1.1)
Ponatinib	134	7430	2.9 (2.4–3.4)	3.5 (2.9–4.3)
Other anti-cancer drugs	9505	1,505,487	1 [Reference]	1 [Reference]

* adjusted for age and sex.

Table S6. Disproportionality analysis of ischemic heart disease submitted for TKIs compared to other anti-cancer drugs.

Ischaemic Heart Disease				
**Drugs**	**Cases**	**Non-cases**	**Crude ROR (95% CI)**	**aROR* (95% CI)**
All TKIs	1565	51,060	3.9 (3.7–4.1)	3.8 (3.6–4.1)
Bosutinib	11	499	2.6 (1.4–4.7	2.5 (1.3–4.8)
Dasatinib	122	14,252	1.0 (0.8–1.2)	1.0 (0.8–1.2)
Imatinib	35	5698	0.7 (0.5–1.0)	0.6 (0.4–0.9)
Nilotinib	1243	23,201	6.7 (6.3–7.1)	6.7 (6.2–7.2)
Ponatinib	154	7410	2.4 (2.0–2.8)	2.9 (2.4–3.5)
Other anti-cancer drugs	11,913	1,503,079	1 [Reference]	1 [Reference]

* adjusted for age and sex.

Table S7. Disproportionality analysis of pulmonary hypertension submitted for TKIs compared to other anti-cancer drugs.

Pulmonary Hypertension				
**Drugs**	**Cases**	**Non-cases**	**Crude ROR (95% CI)**	**aROR * (95% CI)**
All TKIs	175	52,450	3.5 (3.0–4.1)	3.9 (3.2–4.7)
Bosutinib	1	509	n/a	n/a
Dasatinib	113	14,261	8.3 (6.8–10.0)	8.5 (6.8–10.6)
Imatinib	19	5714	3.3 (2.0–5.2)	3.9 (2.4–6.4)
Nilotinib	30	24,414	1.2 (0.8–1.7)	1.1 (0.6–1.7)
Ponatinib	12	7552	1.6 (0.9–2.7)	1.3 (0.6–3.9)
Other anti-cancer drugs	1,422	1,513,570	1 [Reference]	1 [Reference]

* adjusted for age and sex.

Table S8. Disproportionality analysis of *torsade de pointes*/QT prolongation submitted for TKIs compared to other anti-cancer drugs.

***Torsade de pointes*/QT Prolongation**				
**Drugs**	**Cases**	**Non-cases**	**Crude ROR (95% CI)**	**aROR * (95% CI)**
All TKIs	296	52,329	7.3 (6.4–8.3)	6.6 (5.6–7.8)
Bosutinib	1	509	n/a	n/a
Dasatinib	36	14,338	2.7 (1.9–3.8)	2.5 (1.6–3.7)
Imatinib	6	5727	1.1 (0.5–2.5)	0.8 (0.6–2.5)
Nilotinib	248	24,196	12.9 (11.2–14.8)	12.2 (10.3–14.6)
Ponatinib	5	7559	0.7 (0.3–1.7)	0.9 (0.3–2.4)
Other anti-cancer drugs	1178	1,513,814	1 [Reference]	1 [Reference]

* adjusted for age and sex.

Table S9. Table with SMQ "Cardiac Failure" and PTs used.

SMQ: Cardiac Failure		
PT	Acute left ventricular failure	Cor pulmonale acute
	Acute pulmonary oedema	Cor pulmonale chronic
	Acute right ventricular failure	Ejection fraction decreased
	Cardiac asthma	Hepatic congestion
	Cardiac failure	Hepatojugular reflux
	Cardiac failure acute	Left ventricular failure
	Cardiac failure chronic	Low cardiac output syndrome
	Cardiac failure congestive	Neonatal cardiac failure
	Cardiac failure high output	Obstructive shock
	Cardiogenic shock	Pulmonary oedema
	Cardiopulmonary failure	Pulmonary oedema neonatal
	Cardiorenal syndrome	Radiation associated cardiac failure
	Chronic left ventricular failure	Right ventricular ejection fraction decreased
	Chronic right ventricular failure	Right ventricular failure
	Cor pulmonale	Ventricular failure

Table S10. Table with SMQ "Cardiomyopathy" and PTs used.

SMQ: Cardiomyopathy		
PT	Atrial septal defect acquired	Hypertensive cardiomyopathy
	Biopsy heart abnormal	Hypertrophic cardiomyopathy
	Cardiac amyloidosis	Ischaemic cardiomyopathy
	Cardiac hypertrophy	Kearns-Sayre syndrome
	Cardiac sarcoidosis	Metabolic cardiomyopathy
	Cardiac septal hypertrophy	Muscular dystrophy
	Cardiac siderosis	Myocardial calcification
	Cardiomyopathy	Myocardial fibrosis
	Cardiomyopathy acute	Myocardial haemorrhage
	Cardiomyopathy alcoholic	Non-obstructive cardiomyopathy
	Cardiomyopathy neonatal	Peripartum cardiomyopathy
	Cardiotoxicity	Pulmonary arterial wedge pressure increased
	Congestive cardiomyopathy	Restrictive cardiomyopathy
	Cytotoxic cardiomyopathy	Right ventricular ejection fraction decreased
	Diabetic cardiomyopathy	Stress cardiomyopathy
	Ejection fraction abnormal	Tachycardia induced cardiomyopathy
	Ejection fraction decreased	Thyrotoxic cardiomyopathy
	Eosinophilic myocarditis	Ventricular septal defect acquired
	HIV cardiomyopathy	Viral cardiomyopathy

Table S11. Table with SMQ "Hypertension" and PTs used.

SMQ: Hypertension		
PT	Accelerated hypertension	Hypertensive heart disease
	Blood pressure ambulatory increased	Hypertensive nephropathy
	Blood pressure diastolic increased	Labile hypertension
	Blood pressure inadequately controlled	Malignant hypertension
	Blood pressure increased	Malignant hypertensive heart disease
	Blood pressure management	Malignant renal hypertension
	Blood pressure orthostatic increased	Maternal hypertension affecting foetus
	Blood pressure systolic increased	Mean arterial pressure increased
	Diastolic hypertension	Metabolic syndrome
	Eclampsia	Neurogenic hypertension

	Endocrine hypertension	Orthostatic hypertension
	Essential hypertension	Page kidney
	Gestational hypertension	Pre-eclampsia
	HELLP syndrome	Prehypertension
	Hyperaldosteronism	Primary hyperaldosteronism
	Hypertension	Procedural hypertension
	Hypertension neonatal	Renal hypertension
	Hypertensive angiopathy	Renal sympathetic nerve ablation
	Hypertensive cardiomegaly	Renovascular hypertension
	Hypertensive cardiomyopathy	Retinopathy hypertensive
	Hypertensive cerebrovascular disease	Secondary aldosteronism
	Hypertensive crisis	Secondary hypertension
	Hypertensive emergency	Supine hypertension
	Hypertensive encephalopathy	Systolic hypertension
	Hypertensive end-organ damage	Withdrawal hypertension

Table S12. Table with SMQ "Pulmonary hypertension" and PTs used.

**SMQ: Pulmonary Hypertension**		
**PT**	Acute right ventricular failure	Pulmonary valve incompetence
	Cardiac ventriculogram right abnormal	Pulmonary vascular resistance abnormality
	Central venous pressure increased	Pulmonary vein occlusion
	Chronic right ventricular failure	Pulmonary vein stenosis
	Cor pulmonale	Pulmonary veno-occlusive disease
	Cor pulmonale acute	Right atrial dilatation
	Cor pulmonale chronic	Right atrial enlargement
	Portopulmonary hypertension	Right atrial hypertrophy
	Pulmonary arterial hypertension	Right atrial pressure increased
	Pulmonary arterial pressure abnormal	Right ventricular dilatation
	Pulmonary arterial pressure increased	Right ventricular dysfunction
	Pulmonary arterial wedge pressure increased	Right ventricular enlargement
	Pulmonary artery dilatation	Right ventricular failure
	Pulmonary artery thrombosis	Right ventricular heave
	Pulmonary artery wall hypertrophy	Right ventricular hypertension
	Pulmonary capillary haemangiomatosis	Right ventricular hypertrophy
	Pulmonary endarterectomy	Right ventricular systolic pressure increased
	Pulmonary hypertension	Tricuspid valve incompetence
	Pulmonary hypertensive crisis	Vascular resistance pulmonary increased
	Pulmonary tumour thrombotic microangiopathy	

Table S13. Table with SMQ "Ischaemic heart disease" and PTs used.

**SMQ: Ischaemic Heart Disease**		
**PT**	Acute coronary syndrome	Coronary arterial stent insertion
	Acute myocardial infarction	Coronary artery bypass
	Angina unstable	Coronary artery compression
	Blood creatine phosphokinase MB abnormal	Coronary artery disease
	Blood creatine phosphokinase MB increased	Coronary artery dissection
	Coronary artery embolism	Coronary artery insufficiency
	Coronary artery occlusion	Coronary artery restenosis
	Coronary artery reocclusion	Coronary artery stenosis
	Coronary artery thrombosis	Coronary artery surgery
	Coronary bypass thrombosis	Coronary brachytherapy
	Coronary vascular graft occlusion	Coronary bypass stenosis
	Kounis syndrome	Coronary endarterectomy
	Myocardial infarction	Coronary no-reflow phenomenon

	Myocardial necrosis	Coronary ostial stenosis
	Myocardial reperfusion injury	Coronary revascularisation
	Myocardial stunning	Coronary vascular graft stenosis
	Papillary muscle infarction	Dissecting coronary artery aneurysm
	Periprocedural myocardial infarction	ECG signs of myocardial ischaemia
	Post procedural myocardial infarction	External counterpulsation
	Postinfarction angina	Haemorrhage coronary artery
	Silent myocardial infarction	Ischaemic cardiomyopathy
	Troponin I increased	Ischaemic mitral regurgitation
	Troponin increased	Microvascular coronary artery disease
	Troponin T increased	Myocardial hypoxia
	Angina pectoris	Myocardial ischaemia
	Angina unstable	Percutaneous coronary intervention
	Anginal equivalent	Prinzmetal angina
	Arteriosclerosis coronary artery	Stress cardiomyopathy
	Arteriospasm coronary	Subclavian coronary steal syndrome
	Coronary angioplasty	Subendocardial ischaemia

Table S14. Table with SMQ "*Torsade de pointes*/QT prolongation" and PTs used.

**SMQ: *Torsade de pointes*/QT Prolongation**		
**PT**	Electrocardiogram QT interval abnormal	Long QT syndrome congenital
	Electrocardiogram QT prolonged	Torsade de pointes
	Long QT syndrome	Ventricular tachycardia

Table S15. Table with SMQ "Cardiac arrhythmia" and PTs used.

**SMQ: Cardiac Arrhythmia**		
**PT**	Chronotropic incompetence	Withdrawal arrhythmia
	Electrocardiogram repolarisation abnormality	Accelerated idioventricular rhythm
	Electrocardiogram RR interval prolonged	Cardiac fibrillation
	Electrocardiogram U wave inversion	Parasystole
	Electrocardiogram U wave present	Rhythm idioventricular
	Electrocardiogram U-wave abnormality	Torsade de pointes
	Sudden cardiac death	Ventricular arrhythmia
	Arrhythmia	Ventricular extrasystoles
	Heart alternation	Ventricular fibrillation
	Heart rate irregular	Ventricular flutter
	Pacemaker generated arrhythmia	Ventricular parasystole
	Pacemaker syndrome	Ventricular pre-excitation
	Paroxysmal arrhythmia	Ventricular tachyarrhythmia
	Pulseless electrical activity	Ventricular tachycardia
	Reperfusion arrhythmia	

Table S16. Table with SMQ "Embolic and thrombotic events" and PTs used.

**SMQ: Embolic and Thrombotic Events**		
**PT**	Acute aortic syndrome	Pulmonary venous thrombosis
	Acute myocardial infarction	Renal vein embolism
	Amaurosis	Renal vein occlusion
	Amaurosis fugax	Renal vein thrombosis
	Angioplasty	Retinal vein occlusion
	Aortic bypass	Retinal vein thrombosis
	Aortic embolus	SI QIII TIII pattern
	Aortic surgery	Splenic vein occlusion
	Aortic thrombosis	Splenic vein thrombosis
	Aortogram abnormal	Subclavian vein occlusion
	Arterectomy	Subclavian vein thrombosis

	Arterectomy with graft replacement	Superior sagittal sinus thrombosis
	Arterial bypass occlusion	Superior vena cava occlusion
	Arterial bypass operation	Superior vena cava syndrome
	Arterial bypass thrombosis	Thrombophlebitis
	Arterial graft	Thrombophlebitis migrans
	Arterial occlusive disease	Thrombophlebitis neonatal
	Arterial stent insertion	Thrombophlebitis superficial
	Arterial therapeutic procedure	Thrombosed varicose vein
	Arterial thrombosis	Thrombosis corpora cavernosa
	Arteriogram abnormal	Transverse sinus thrombosis
	Arteriogram carotid abnormal	Vascular graft
	Arteriotomy	Vena cava embolism
	Atherectomy	Vena cava filter insertion
	Atherosclerotic plaque rupture	Vena cava filter removal
	Atrial appendage closure	Vena cava thrombosis
	Basal ganglia infarction	Venogram abnormal
	Basilar artery occlusion	Venoocclusive disease
	Basilar artery thrombosis	Venoocclusive liver disease
	Blindness transient	Venous angioplasty
	Brachiocephalic artery occlusion	Venous occlusion
	Capsular warning syndrome	Venous operation
	Carotid angioplasty	Venous recanalisation
	Carotid arterial embolus	Venous repair
	Carotid artery bypass	Venous stent insertion
	Carotid artery occlusion	Venous thrombosis
	Carotid artery stent insertion	Venous thrombosis in pregnancy
	Carotid artery thrombosis	Venous thrombosis limb
	Carotid endarterectomy	Venous thrombosis neonatal
	Cerebellar artery occlusion	Visceral venous thrombosis
	Cerebellar artery thrombosis	Administration site thrombosis
	Cerebellar embolism	Adrenal thrombosis
	Cerebral artery embolism	Angiogram abnormal
	Cerebral artery occlusion	Angiogram cerebral abnormal
	Cerebral artery thrombosis	Angiogram peripheral abnormal
	Cerebral hypoperfusion	Angioplasty
	Cerebral vascular occlusion	Application site thrombosis
	Cerebrovascular insufficiency	Arteriovenous fistula occlusion
	Cerebrovascular stenosis	Arteriovenous fistula thrombosis
	Coeliac artery occlusion	Arteriovenous graft thrombosis
	Coronary angioplasty	Artificial blood vessel occlusion
	Coronary arterial stent insertion	Atrial thrombosis
	Coronary artery bypass	Basal ganglia stroke
	Coronary artery embolism	Bone infarction
	Coronary artery occlusion	Brain stem embolism
	Coronary artery reocclusion	Brain stem infarction
	Coronary artery surgery	Brain stem stroke
	Coronary artery thrombosis	Brain stem thrombosis
	Coronary endarterectomy	Cardiac ventricular thrombosis
	Coronary revascularisation	Catheter site thrombosis
	Coronary vascular graft occlusion	Cerebellar embolism
	Embolia cutis medicamentosa	Cerebellar infarction
	Embolism	Cerebral congestion
	Embolism arterial	Cerebral infarction
	Endarterectomy	Cerebral infarction foetal
	Femoral artery embolism	Cerebral ischaemia
	Hepatic artery embolism	Cerebral microembolism
	Hepatic artery occlusion	Cerebral septic infarct
	Hepatic artery thrombosis	Cerebral thrombosis
	Hypothenar hammer syndrome	Cerebral vascular occlusion
	Iliac artery embolism	Cerebrospinal thrombotic tamponade
	Iliac artery occlusion	Cerebrovascular accident
	Intra-aortic balloon placement	Cerebrovascular accident prophylaxis
	Intraoperative cerebral artery occlusion	Cerebrovascular disorder
	Ischaemic cerebral infarction	Cerebrovascular operation
	Ischaemic stroke	Choroidal infarction
	Lacunar infarction	Collateral circulation
	Leriche syndrome	Coronary angioplasty

	Mesenteric arterial occlusion	Coronary artery thrombosis
	Mesenteric arteriosclerosis	Coronary bypass thrombosis
	Mesenteric artery embolism	Device embolisation
	Mesenteric artery stenosis	Device occlusion
	Mesenteric artery stent insertion	Device related thrombosis
	Mesenteric artery thrombosis	Diplegia
	Myocardial infarction	Directional Doppler flow tests abnormal
	Myocardial necrosis	Disseminated intravascular coagulation
	Papillary muscle infarction	Disseminated intravascular coagulation in newborn
	Penile artery occlusion	Embolic cerebral infarction
	Percutaneous coronary intervention	Embolic pneumonia
	Peripheral arterial occlusive disease	Embolic stroke
	Peripheral arterial reocclusion	Embolism
	Peripheral artery angioplasty	Foetal cerebrovascular disorder
	Peripheral artery bypass	Graft thrombosis
	Peripheral artery occlusion	Haemorrhagic adrenal infarction
	Peripheral artery stent insertion	Haemorrhagic cerebral infarction
	PT Peripheral artery thrombosis	Haemorrhagic infarction
	Peripheral embolism	Haemorrhagic stroke
	Peripheral endarterectomy	Haemorrhagic transformation stroke
	Popliteal artery entrapment syndrome	Haemorrhoids thrombosed
	Post procedural myocardial infarction	Hemiparesis
	Postinfarction angina	Hemiplegia
	Precerebral artery occlusion	Heparin-induced thrombocytopenia
	Precerebral artery thrombosis	Hepatic infarction
	Profundaplasty	Hepatic vascular thrombosis
	Pulmonary artery occlusion	Implant site thrombosis
	Pulmonary artery therapeutic procedure	Incision site vessel occlusion
	PT Pulmonary artery thrombosis	Infarction
	Pulmonary endarterectomy	Infusion site thrombosis
	Pulmonary tumour thrombotic microangiopathy	Injection site thrombosis
	Renal artery angioplasty	Inner ear infarction
	Renal artery occlusion	Instillation site thrombosis
	Renal artery thrombosis	Intestinal infarction
	Renal embolism	Intracardiac mass
	Retinal artery embolism	Intracardiac thrombus
	Retinal artery occlusion	Medical device site thrombosis
	Retinal artery thrombosis	Mesenteric arterial occlusion
	Silent myocardial infarction	Mesenteric vascular insufficiency
	Spinal artery embolism	Mesenteric vascular occlusion
	Spinal artery thrombosis	Mesenteric venous occlusion
	Splenic artery thrombosis	Microembolism
	Splenic embolism	Monoparesis
	Stress cardiomyopathy	Monoplegia
	Stroke in evolution	Optic nerve infarction
	Subclavian artery embolism	Pancreatic infarction
	Subclavian artery occlusion	Paradoxical embolism
	Subclavian artery thrombosis	Paraneoplastic thrombosis
	Superior mesenteric artery syndrome	Paraparesis
	Thromboembolectomy	Paraplegia
	Thrombotic microangiopathy	Paresis
	Thrombotic thrombocytopenic purpura	Peripheral revascularisation
	Transient ischaemic attack	Pituitary infarction
	Truncus coeliacus thrombosis	Placental infarction
	Vascular pseudoaneurysm thrombosis	Pneumatic compression therapy
	Vertebral artery occlusion	Portal shunt
	Vertebral artery thrombosis	Portal shunt procedure
	Visual acuity reduced transiently	Post procedural stroke
	Axillary vein thrombosis	Postpartum thrombosis
	Brachiocephalic vein occlusion	Prosthetic vessel implantation
	Brachiocephalic vein thrombosis	Quadriparesis
	Budd-Chiari syndrome	Quadriplegia
	Catheterisation venous	Renal artery angioplasty
	Cavernous sinus thrombosis	Renal infarct
	Central venous catheterisation	Renal vascular thrombosis
	Cerebral venous thrombosis	Retinal infarction
	Compression garment application	Retinal vascular thrombosis

	Deep vein thrombosis	Shunt occlusion
	Deep vein thrombosis postoperative	Shunt thrombosis
	Embolism venous	Silent myocardial infarction
	Hepatic vein embolism	Spinal cord infarction
	Hepatic vein occlusion	Splenic infarction
	Hepatic vein thrombosis	Splenic thrombosis
	Homans' sign positive	Stoma site thrombosis
	Iliac vein occlusion	Stroke in evolution
	Inferior vena cava syndrome	Surgical vascular shunt
	Inferior vena caval occlusion	Testicular infarction
	Intracranial venous sinus thrombosis	Thalamic infarction
	Jugular vein occlusion	Thrombectomy
	Jugular vein thrombosis	Thromboangiitis obliterans
	Mahler sign	Thrombolysis
	May-Thurner syndrome	Thrombosis
	Mesenteric vein thrombosis	Thrombosis in device
	Mesenteric venous occlusion	Thrombosis mesenteric vessel
	Obstetrical pulmonary embolism	Thrombosis prophylaxis
	Obstructive shock	Thrombotic cerebral infarction
	Ophthalmic vein thrombosis	Thrombotic stroke
	Ovarian vein thrombosis	Thyroid infarction
	Paget-Schroetter syndrome	Tumour embolism
	Pelvic venous thrombosis	Tumour thrombosis
	Penile vein thrombosis	Ultrasonic angiogram abnormal
	Phlebectomy	Ultrasound Doppler abnormal
	Portal vein cavernous transformation	Umbilical cord occlusion
	Portal vein occlusion	Umbilical cord thrombosis
	Portal vein thrombosis	Vaccination site thrombosis
	Portosplenomesenteric venous thrombosis	Vascular access site thrombosis
	Post procedural pulmonary embolism	Vascular graft
	Post thrombotic syndrome	Vascular graft occlusion
	Postoperative thrombosis	Vascular graft thrombosis
	Postpartum venous thrombosis	Vascular operation
	Pulmonary embolism	Vascular stent insertion
	Pulmonary infarction	Vascular stent occlusion
	Pulmonary microemboli	Vascular stent thrombosis
	Pulmonary oil microembolism	Vasodilation procedure
	Pulmonary thrombosis	Vessel puncture site occlusion
	Pulmonary vein occlusion	Vessel puncture site thrombosis
	Pulmonary veno-occlusive disease	Visual midline shift syndrome

© 2020 by the authors. Licensee MDPI, Basel, Switzerland. This article is an open access article distributed under the terms and conditions of the Creative Commons Attribution (CC BY) license (http://creativecommons.org/licenses/by/4.0/).
